# Shrinkage of hepatocellular carcinoma after radiofrequency ablation following transcatheter arterial chemoembolization: Analysis of contributing factors

**DOI:** 10.1371/journal.pone.0210667

**Published:** 2019-02-28

**Authors:** Mi Hye Yu, Young Jun Kim, Hee Sun Park, Sung Il Jung, Hae Jeong Jeon

**Affiliations:** Department of Radiology, Konkuk University Medical Center, Konkuk University School of Medicine, Seoul, Korea; Texas A&M University, UNITED STATES

## Abstract

**Objective:**

This study was conducted to investigate tumor shrinkage and influencing factors in patients with hepatocellular carcinoma (HCC) from radiofrequency (RF) ablation following transcatheter arterial chemoembolization (TACE).

**Methods:**

A total of 222 patients underwent combined sequential treatment of TACE and RF ablation for HCC at our institution between 2008 and 2014. Of those, 86 patients (men, 68; women, 18) who achieved compact iodized oil tagging and complete ablation were included for this retrospective study. We measured three-dimensional tumor diameters and calculated tumor volumes on pre-treatment CT/MRI and follow-up CT scans performed post-TACE, post-ablation, and 1 month post-treatment, respectively. To compare periodically generated tumor diameters and volumes, repeated measures analysis of variance (ANOVA) was applied. Multiple linear regression analysis was performed to identify factors impacting tumor shrinkage after RF ablation.

**Results:**

Diameters and volumes of HCCs declined significantly in the immediate aftermath of RF ablation (i.e., between post-TACE and post-ablation CT scans) (*p* < 0.001, for both). Mean reduction rates in tumor diameter and volume immediately after RF ablation were 18.2 ± 9.1% and 44.4 ± 14.6%, respectively. Of note, tumors of left hepatic lobe and in subphrenic or perivascular locations showed lower rates of post-ablative volume reduction than those in counterpart locations (*p =* 0.002, 0.046, 0.024, respectively). Tumor size and liver function did not influence tumor shrinkage after RF ablation.

**Conclusion:**

In patients with HCC, significant tumor shrinkage occurs immediately after RF ablation. The degree of shrinkage in response to ablative treatment seems to vary by tumor location.

## Introduction

Radiofrequency (RF) ablation is a common curative treatment for hepatocellular carcinoma (HCC) [1]. It is a locally applied thermal ablation technique intended to destroy tumor using heat [2]. Thermal ablation induces protein denaturation and dehydration, as well as contraction of collagen and tissue shrinkage [3,4]. In clinical and experimental studies, it has been noted that ablated tissues undergo involution [5–8]. However, it is not possible to clinically identify tumor shrinkage induced by RF ablation, because in CT studies, ablated tumor is indistinguishable from surrounding ablated tissue. Indeed, studies [9–11] conducted to date in this regard have measured ablation zones (including both tumor and surrounding ablated tissue), rarely measuring ablated tumor directly.

RF ablation combined with transcatheter arterial chemoembolization (TACE) has been lauded for its superior efficacy in the treatment of HCC, compared with RF ablation only [12–15]. This combined therapy provides a unique opportunity for the accurate assessment of tumor shrinkage itself on post-ablative CT. Iodized oil deposited via TACE is consequently visible as a high-attenuation nodule, easily discriminated from surrounding the low-attenuation ablation zone on post-ablative CT images [13,16]. One may then directly measure tumor diameters in both pre- and post-ablative CT scans to assess changes in size and volume.

We presumed that significant tumor shrinkage occurs immediately after RF ablation and various clinical and imaging variables in this setting contribute to significant tumor shrinkage immediately after RF ablation, thus we analyzed data from patients undergoing RF ablation following TACE oil tagging of HCC. Therefore, the purpose of this study was to investigate the nature of dimensional and volumetric changes achieved through RF ablation of HCC and to identify factors correlating with post-ablative tumor shrinkage.

## Materials and methods

This study was approved by our institutional review board (Konkuk University Medical Center, KUH1140126). Due to its retrospective design, written informed consent was waived. We regularly adhered to all proposed standard terminology and reporting criteria for image-guided tumor ablation in the course of our work [17].

### Study population

**Fig 1:**
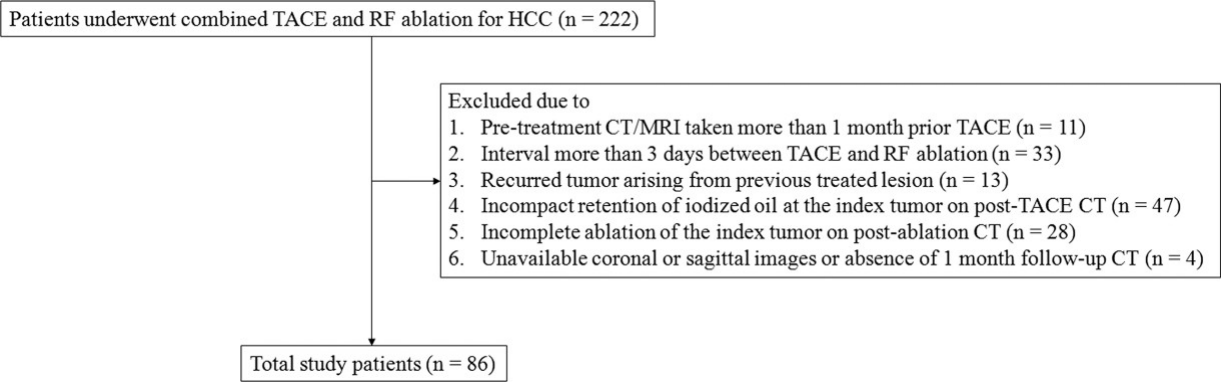
Flow chart of patient selection. TACE = transcatheter arterial chemoembolization, RF = radiofrequency, HCC = hepatocellular carcinoma.

Between January 2008 and December 2014, 222 consecutive patients with HCC received a combined, sequential regimen of TACE and RF ablation at our institution. The therapeutic strategies were addressed for the patients who refused surgical resection or was not indicated for transplantation as a curative treatment by a multidisciplinary tumor board. Ultimately, 86 patients (men, 68; women, 18) of mean age 58.0 ± 9.87 years (range, 33–77 years) qualified for study, given the following inclusion criteria: 1) pre-treatment CT or MRI within 1 month prior to TACE; 2) sequential TACE and RF ablation within 0–3 days; 3) *de novo* index tumor (no previously treated persistent/ recurrent disease); 4) iodized oil concentrated compactly along index tumor on post-TACE CT; 5) complete ablation of index tumor on post-ablative CT, with ≥5-mm safety margin[18]; and 6) multiplanar reformation images (coronal or sagittal) available for three-dimensional (3-D) tumor measurements. A flow chart of patient selection is shown in Fig 1.

Each diagnosis of HCC was established at time of treatment using pre-treatment baseline CT or MRI studies done prior to TACE (within 1 month) and as stipulated by the American Association for the Study of Liver Diseases (AASLD) [19]. To accurately determine tumor margins, iodized oil must be retained and concentrated at its borders. Thus, we excluded those tumors showing poor or no iodized oil retention following TACE procedures. Tumors incompletely ablated were also excluded, owing to expectedly skewed rates of post-ablative volume change. In patients with multiple HCCs, only the largest eligible tumor was studied.

### Combination treatment protocol for hepatocellular carcinoma

**Fig 2:**
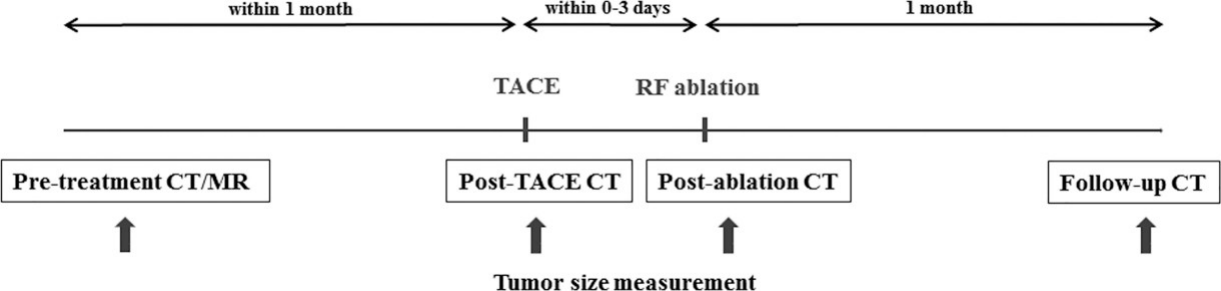
Study algorithm for patients with hepatocellular carcinoma. TACE = transcatheter arterial chemoembolization, RF = radiofrequency.

Our institutional protocol for combination TACE/RF ablation treatment of patients with HCC is described herein and is shown as a schematic in Fig 2.

TACE treatments were undertaken on an inpatient basis using an interventional procedure room equipped with a commercially available digital subtraction angiography system (Axiom Artis dBA; Siemens Healthcare, Erlangen, Germany). Two experienced interventional radiologists (17 and 13 years, respectively) performed all TACE procedures. Upon completing celiac and superior mesenteric arteriography, hepatic artery angiography was performed using a 5-Fr catheter (Cook Medical, Bloomington, IN, USA). Right or left hepatic artery was then selectively catheterized (3-Fr MicroFerret; Cook Medical) to locate a tumor-directed feeder artery. Selective embolization was carried out thereafter, injecting a mixed emulsion of iodized oil (Lipiodol; Andre Guerbet, Aulnay-sous-Bois, France) and doxorubicin hydrochloride (Adriamycin RDF; Ildong Pharmaceutical, Seoul, Korea). Embolization continued until arterial flow stagnated and/or iodized oil was visualized within portal branch vessels. Gelatin sponge particles of 1–2 mm diameter (Gelfoam; Upjohn, Kalamazoo, MI, USA) were also infused. Once accomplished, angiography was again performed to assess the extent of vascular compromise and check for any residual tumor staining. Post-TACE CT obtained the next day served to verify that oil retention by tumor was adequate.

Percutaneous RF ablation took place within 0–3 days after TACE, performed by one of two radiologists (YJK and HSP with 18 and 10 years of experience in this setting, respectively). All procedures routinely involved local anesthesia with conscious sedation; both ultrasound and fluoroscopy guidance; and use of a 15-G or 17-G monopolar, internally cooled electrode bearing a 2–3 cm active tip (VIVA; STARmed, Goyang, Korea) and powered by a 200W generator (VIVA; STARmed). We used a 2-cm tip for smaller tumors (1–2 cm), reserving the 3-cm tip for larger-sized lesions. Depending on tumor size and configuration, single or multiple overlapping ablations were executed. The energy deposition algorithm applied reflected manufacturer’s recommendations. Ablation was terminated once an entire tumor and surrounding hepatic tissue margins fell within in the ultrasound echogenic zone. To prevent bleeding or tract seeding, the electrode path was also cauterized during retraction [20]. Post-ablation CT was undertaken in the immediate aftermath to check for related complications (such as bleeding) and to gauge technical success. Each patient was then subjected to a multiphasic liver CT 1 month after RF ablation as the initial follow-up assessment of tumor remission.

### Scan techniques

All CT examinations entailed use of a 64-MDCT (Somatom Definition [Siemens Healthcare]; LightSpeed VCT [GE Healthcare, Chicago, IL, USA]). The respective scanning parameters used for the two 64-MDCT systems were as follows: detector collimation, 64 × 0.6 mm and 64 × 0.625 mm; pitch, 0.984; and rotation time, 0.5 second. The reference tube current was set at 250 and 200 mAs at 120 kVp, with automated dose modulation. Axial images were reconstructed at section thickness /reconstruction intervals of 3 mm/3 mm (Somatom Definition) and 3.75 mm/3.75 mm (LightSpeed VCT). Coronal images were similarly reconstructed (3 mm/3 mm) using portal venous phase scan.

Pre-treatment, post-ablation, and 1-month follow-up assessments entailed multiphasic liver CT, providing unenhanced, late arterial, portal, and equilibrium phases. A total of 370 mg I/mL of iodinated contrast medium, iopromide (Ultravist 370; Bayer Healthcare, Berlin, Germany), was administered via power injector for 30 seconds at a dose of 1.5 mL/kg (555 mg I/kg) body weight, followed by injection of normal saline solution (30–40 mL). Late arterial, portal venous, and equilibrium-phase images were obtained at 25 sec, 70 sec, and 180 sec after the start of contrast administration. Post-TACE CT consisted of a pre-contrast scan only, without iodized contrast medium.

In nine patients who lacked pre-treatment CT scans, we used MRI scans for image analysis instead of CT scans. Liver MRI was performed via 1.5-T (n = 5, Signa HDxt; GE Healthcare) or 3-T (n = 4, Magnetom Skyra; Siemens Healthcare) superconducting system, using a 32-channel phased-array coil. Dynamic 3-D fat-saturated T1-weighed sequences were obtained after gadoxetic acid (Primovist; Bayer Healthcare) administration. Axial and coronal images were scanned at spatial resolutions of 1.1–1.8 mm and 2.7- to 5.2-mm section thickness in hepatobiliary phase, 20 min after the start of contrast medium injection.

### Image analysis

#### Tumor measurement

**Fig 3:**
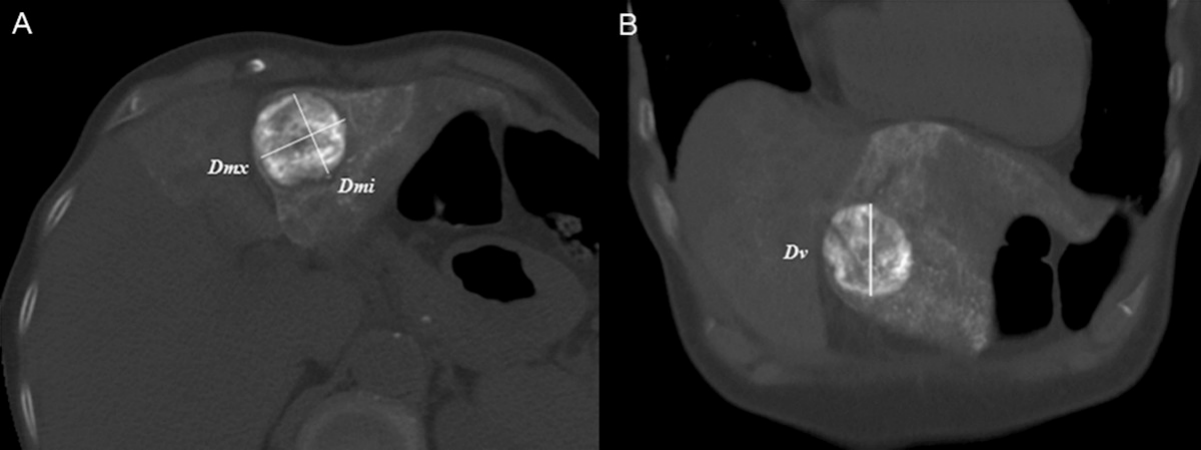
Measurement of three-dimensional (3-D) tumor diameters. Axial (a) and coronal (b) CT images best depicting index tumor serving in 3-D diameter measurements as follows: maximum diameter (Dmx) and perpendicular minimum diameter (Dmi) in axial image (a), and vertical diameter (Dv) in coronal image (b). Note: a preset bone window setting (width, 3000 HU; level, 500 HU) is applied to avoid beam-hardening artifact due to iodized oil retention by index tumors or surrounding parenchyma.

Two clinically experienced abdominal radiologists (YJK and MHY) with years of cumulative expertise (18 and 7 years, respectively) measured 3-D diameters of each index tumor using pre-treatment CT/MRI and CT scans done post-TACE, post-ablation, and at the 1-month follow-up point. Imaging reviews were facilitated by PACS software (Centricity RA1000; GE Healthcare), shown on monitors at 2048 × 2560 spatial resolution. For precision in measurement, all images were displayed at one-by-one setting, and the PACS magnification function was used. Two reviewers initially screened images, selecting (by consensus) those that best depicted index tumors in axial, coronal, or sagittal planes. Actual 3-D diameters were generated from axial images (maximum diameter [D*mx*] and perpendicular minimum diameter [D*mi*]) and coronal or sagittal views (vertical [craniocaudal] diameter [D*v*]) (Fig 3).

Measurements based on pre-treatment CT scans were obtained at a preset soft tissue window setting (width, 350 HU; level, 50 HU). To avoid the beam-hardening artifact due to iodized oil retention by index tumors or adjacent parenchyma, a preset bone window setting (width, 3000 HU; level, 500 HU) was adapted for post-TACE, post-ablation, and follow-up CT scans. In nine patients lacking pre-treatment CT scans as stipulated (within 1 month before TACE), MRI served for image analysis. Measurements were obtained from axial and coronal images in hepatobiliary phase of scanning, owing to the high contrast resolution between index tumor and liver parenchyma. Tumor volume was ultimately calculated by applying above 3-D diameters to the following equation:

$$Volume=\frac{\pi }{6}\times Dmx\times Dmi\times Dv$$

Rates of tumor diameter and volume reduction over time, from pre-treatment CT/MRI to CT scans performed post-TACE, post-ablation, and in follow-up, were calculated using the following formula:

$$Reduction\;rate=\frac{baseline\;tumor\;value-follow\;up\;tumor\;value}{baseline\;tumor\;value}\times 100$$

#### Factors impacting tumor shrinkage

To identify factors of potential influence in tumor shrinkage after RF ablation, we evaluated several clinical or laboratory parameters and some imaging features, including the presence of portal hypertension, tumor size, and tumor location. With the presumption that portosystemic collaterals are surrogate markers for portal hypertension [21], pre-treatment CT/MR images were duly screened for their presence. We also established several analytic variables in terms of tumor location, namely lobar (right vs left hepatic lobe), subcapsular (vs non-subcapsular), subphrenic (vs non-subphrenic), and perivascular (vs non-perivascular) sites. Subcapsular location was marked by hepatic capsular abutment of index tumor in axial or coronal images [22,23]. Subphrenic location similarly was equated with diaphragmatic abutment of index tumor in axial or coronal images [24,25]. Contact between index tumor and any first- or second-degree branches of portal vein or branches of hepatic vein ≥3 mm in axial diameter qualified as perivascular location [26–28].

#### Therapeutic outcomes

Local tumor progression, overall survival rate, and complications after combined TACE and RF ablation treatment were assessed. Local tumor progression is defined as the newly developed tumor at the margin of the ablation zone on follow-up images. Overall survival rate was analyzed using the interval between the RF ablation and either the death or the last visit to the hospital. Major and minor complications were assessed in accordance with the Society of Interventional Radiology guideline [29].

### Statistical analysis

Tumor diameters and volumes and rates of shrinkage were individually expressed as mean ± standard deviation (SD). To assess dimensional/volumetric differences in tumors at various imaging time points, including pre-treatment, post-TACE, post-ablation, and 1 month post-treatment, repeated measures analysis of variance (ANOVA) was performed, applying the Bonferroni adjustment for multiple comparisons.

Student’s *t*-test and the Kruskal-Wallis test were used to compare post-ablative rates of tumor volume reduction according to clinical and imaging features. The following patient variables were analyzed: age, gender, Child-Pugh score, serum alpha-fetoprotein, time interval (days) between TACE and RF ablation, portal hypertension (+/-), tumor size, and assorted tumor locations (lobar, subcapsular, subphrenic, or perivascular). Factors independently associated with post-ablative tumor shrinkage were identified by multiple linear regression method. The cumulative local tumor progression rates and overall survival rates were estimated by using the method of Kaplan-Meier. All computations relied on standard software (SPSS v17.0; SPSS Inc, Chicago, IL, USA), setting statistical significance at *p* < 0.05.

## Results

Characteristics of the patient population are summarized in Table 1.

**Table 1:** Characteristics of study population.

Characteristic	Value
No. of patients	n = 86
Male: female	68:18
Age (yr)	mean 58.0 ± 9.87 yr (range 33–77 yr)
Etiology of liver disease	
Hepatitis B virus	61
Hepatitis C virus	14
Hepatitis B and C viruses	1
Alcohol	7
Cryptogenic	3
Child-Pugh score	
5	64
6	11
7	5
8	4
9	2
Serum AFP (ng/ml), > 100: ≤100	15:71
MELD score	8.7 ± 2.6 (range 6–19)
MELD-Na score	10.3 ± 2.9 (range 6–20)
ECOG performance status	
0	86
BCLC stage	
0	48
A	35
B	3
Interval between TACE and RF ablation (day)	
Mean ± SD (range)	1.64 ± 0.78 day (range 0–3 day)
0	1
1	44
2	26
3	15
Presence of portal hypertension	
Yes: No	46:40
Tumor size (cm)	
Mean ± SD (range)	1.94 ± 0.86 cm (range 1.00–6.05 cm)
1-2cm	56
2-3cm	22
3 or larger	8
Tumor location (Couinaud segment)	
Segment II	2
Segment III	6
Segment IV	6
Segment V	14
Segment VI	15
Segment VII	14
Segment VIII	29

AFP = alpha-fetoprotein, MELD = model for end-stage liver disease, ECOG = Eastern Cooperative Oncology Group, BCLC = Barcelona Clinic Liver Cancer, TACE = transcatheter arterial chemoembolization, RF = radiofrequency

A total of 86 patents (men, 68; women, 18) of mean age 58.0 ± 9.87 years (range, 33–77 years) met our eligibility criteria and qualified for study. Mean tumor size was 1.94 ± 0.86 cm (range, 1.00–6.05 cm). The mean interval between pre-treatment CT/MRI and TACE procedures was 11.72 ± 9.68 days (range, 0–30 days), with a mean of 1.64 ± 0.78 days (range, 0–3 days) between TACE and RF ablation.

Diameters (3-D) and volumes of treated tumors and reduction rates achieved are recorded in Table 2.

**Table 2:** Three-dimensional diameters and volumes of hepatocellular carcinomas and reduction rates achieved by radiofrequency ablation after transcatheter arterial chemoembolization.

	Pre-treatment CT/MR	Post-TACE CT	Post-ablation CT	Follow-up CT
Diameters				
D*mx* (mm)	18.8 ± 8.4	18.6 ± 8.3	15.3 ± 6.7	15.1 ± 6.8
Reduction rate (%)	0.8 ± 12.2		17.1 ± 8.1*	1.0 ± 5.8
D*mi* (mm)	16.2 ± 7.9	15.6 ± 7.4	12.7 ± 6.3	12.5 ± 6.1
Reduction rate (%)	2.3 ± 14.1		18.2 ± 9.8*	0.8 ± 7.8
D*v* (mm)	17.4 ± 8.5	17.1 ± 7.3	13.8 ± 6.2	13.6 ± 5.8
Reduction rate (%)	-1.6 ± 16.1		19.2 ± 9.4*	0.7 ± 7.4
Volume				
Volume (mm^3^)	5118.7 ± 12342.7	4493.9 ± 9296.5	2517.6 ± 5506.7	2377.0 ± 5068.6
Reduction rate (%)	- 0.7 ± 34.5		44.4 ±14.6*	2.3 ± 12.9

D*mx =* maximum diameter measured on axial image, D*m =* minimum diameter measured on axial image, D*v =* vertical diameter measured on coronal image, TACE = transcatheter arterial chemoembolization

* statistically significant (*p* < 0.05).

**Fig 4:**
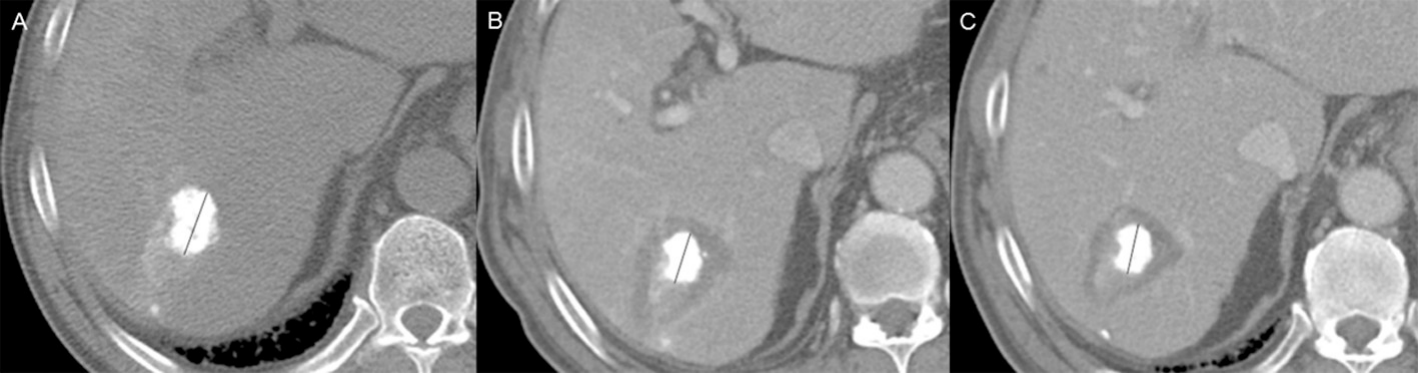
46 year-old man with hepatocellular carcinoma. (a) After transcatheter arterial chemoembolization (TACE), concentrated iodized oil delineates tumor on post-TACE CT (longitudinal tumor diameter, 27.8 mm). (b) Following completion of sequential radiofrequency (RF) ablation, low-attenuation ablation zone encircles index tumor. Tumor size diminished on post-ablation CT (decline in longitudinal tumor diameter: 27.8 mm → 20.7mm; 25.5% post-ablative reduction rate) for post-ablative volume reduction rate of 60.1%. (c) Stability of tumor confirmed by follow-up CT scan 1 month after RF ablation (20.7 mm → 20.4 mm).

There were statistically significant mean rates of decline in diameters (18.2 ± 9.1%; range, 11.4–44.8%) and volumes (44.4 ± 14.6%; range, 0.8–73.7%) of tumors during the time between post-TACE and post-ablative CT scans (*p* < 0.001, both) (Table 2, Fig 4).

However, these parameters did not differ significantly in periods between pre-treatment CT/MRI and post-ablative CT scans or between post-ablative and 1-month follow-up CT scans (*p* > 0.05).

Relations between post-ablative rates of tumor volume reduction and corresponding clinical or imaging features in patients with HCC are summarized in Table 3.

**Table 3:** Relations between clinical or imaging features and volume reduction rates of hepatocellular carcinoma after radiofrequency ablation.

Features	Number	Volume reduction rate (%)	*P* value
Age			
> 60	44	46.1 ± 12.7	*0*.*271*
≤ 60	42	42.6 ± 16.4	
Gender			
Male	68	45.7 ± 13.3	*0*.*190*
Female	18	39.5 ± 18.4	
Child-Pugh score classification			
A	75	45.1 ± 14.1	*0*.*247*
B	11	39.6 ± 18.1	
Serum AFP			
≤100	71	45.4 ± 14.5	*0*.*190*
> 100	15	39.7 ± 14.8	
Interval between TACE and RF ablation			
0 or 1	45	45.4 ± 13.0	*0*.*516*
2 or 3	41	43.3 ± 16.3	
Presence of portal hypertension			
Yes	46	45.7 ± 13.6	*0*.*442*
No	40	43.3 ± 15.5	
Tumor size			
1-2cm	56	42.7 ± 15.5	*0*.*134*
2-3cm	22	49.3 ± 11.8	
3cm or greater	8	48.6 ± 12.4	
Lobar location			
Right lobe	72	46.5 ± 13.1	***0*.*002***
Left lobe	14	33.7 ± 17.8	
Subcapsular location			
Yes	29	44.9 ± 15.8	*0*.*812*
No	57	44.1 ± 14.2	
Subphrenic location			
Yes	12	36. 6 ± 15.0	***0*.*046***
No	74	45.7 ± 14.3	
Perivascular location			
Yes	19	37.8 ± 15.5	***0*.*024***
No	67	46.3 ± 14.0	

AFP = alpha-fetoprotein, TACE = transcatheter arterial chemoembolization, RF = radiofrequency

Such rates were significantly less in left (vs right) lobar tumors (33.7% vs 46.5%; *p* = 0.002), in subphrenic (vs non-subphrenic) tumors (36.6% vs 45.7%; *p* = 0.046), and in perivascular (vs non-perivascular) tumors (37.8% vs 46.3%; *p* = 0.024). However, reductions in tumor volume after RF ablation did not differ significantly when analyzed by Child-Pugh score, tumor size, subscapular tumor location, or presence/absence of portal hypertension.

Results of multiple linear regression analysis are presented in Table 4.

**Table 4:** Multiple linear regression analysis of hepatocellular carcinoma volume reduction rate after radiofrequency ablation.

Variables	Regression Coefficient (ß)	Standard Error	*P* value
Left lobe tumor location	-11.269	3.905	0.005
Subphrenic tumor location	-11.363	4.215	0.009
Perivascular tumor location	-9.116	3.558	0.012

Again, left lobar, subphrenic, and perivascular tumor locations emerged as variables independently associated with diminished rates of tumor volume reduction after RF ablation (*p* = 0.005, *p* = 0.009, *p* = 0.012, respectively).

**Fig 5:**
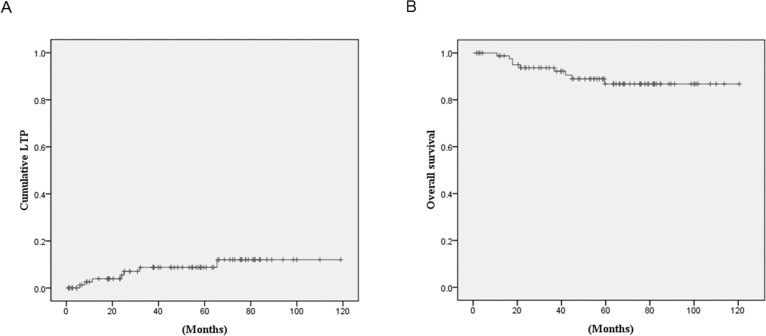
Cumulative local tumor progression rate and overall survival rate curves. (a) Cumulative local tumor progression rates in the overall data. (b) Overall survival rates in the overall data. LTP = local tumor progression.

Local tumor progression was found in 7 (8.2%) of 86 HCCs during the follow-up period (mean follow up: 47.0 ± 30.3 months; range: 0.9–119 months). The cumulative local tumor progression rates at 1, 3, and 5 years were 3.9%, 8.8%, and 8.8%, respectively (Fig 5A). The overall survival rates at 1, 3, and 5 years were 98.8%, 93.7%, and 86.8%, respectively (Fig 5B).

There were treatment-related deaths and no major complications developed. There were 16 minor complications (18.6%): post-ablation syndrome (n = 10), diaphragmatic thermal injury (n = 3), perihepatic hematoma (n = 2), and pleural effusion (n = 1). All patients with minor complications were treated with analgesics. None of the patients experienced long-term hepatic dysfunction or hepatic failure after the procedure.

## Discussion

Results of the present study demonstrate that significant declines in tumor volume occur immediately after RF ablation performed in patients with HCC, regardless of baseline tumor size or liver function status. Mean diameter and volume reduction rates immediately following RF ablation were 18.2 ± 9.1% and 44.4 ± 14.6%, respectively. In addition, the degree of tumor shrinkage observed after RF ablation differed significantly according to tumor location, with left lobar, subphrenic, and perivascular locations showing significantly less tumor shrinkage than tumors at counterpart locations.

In an *ex vivo* study conducted by Brace et al. [6], ablative tissue contraction was investigated in liver and lung tissues during RF and microwave ablation. They reported hepatic tissue contraction of 2.9–4.8 mm (15–31%) after RF ablation, with more prominent shrinkage manifested in the peripheral coagulation zone. A clinical study pursued by Ganguli et al. [5] similarly confirmed a 5.4 mm (21%) mean decline in renal tumor diameters immediately after RF ablation. In this instance, it was possible to assess post-ablative tumor shrinkage through changes in contour rather than direct visualization, because most of the renal tumors treated were exophytic in nature. Our patients with HCC likewise showed a mean decline of 3.2 mm (18.2%) immediately after RF ablation, aligning with the assorted investigations above. Recently, Lee et al. [30] also assessed degrees of RF- and microwave-induced tissue shrinkage in liver tumors, while curiously reporting a trend (inconsistent with earlier data) toward minimal tissue swelling (1.3%) after RF ablation. Because the outlines of ablated tumors were still visible within treated zones in post-ablation MRI studies, they directly measured post-ablative tumor size as well. Unfortunately, this approach succeeded in only about 40% (18/44) of the patients studied, the few tumors (n = 18) evaluated and the wide variation in tumor contraction rate (1.31 ± 13.2%) constituting distinct drawbacks.

To prevent local tumor progression after RF ablation, complete tumor ablation is imperative, and assessment of treated margins is therefore crucial [18,31,32]. In current clinical practice, pre- and post-ablative images are compared side-by-side, using hepatic vessels and liver surface as landmarks to assess ablative margins [33]. Various techniques, such as image fusion, rigid or non-rigid image registration, and quantitative assessment, have been advanced during the past decade to improve post-ablative margin assessment [34–37]. However, tumor shrinkage following RF ablation (as shown herein) has not been addressed in any of the studies reported to date. Because such tumor shrinkage is problematic, leading to underestimation of original ablative zones, it should be considered in studies going forward.

The degree of tumor shrinkage after RF ablation may be impacted by tumor location. Heat-sink effect is a known phenomenon whereby blood flow draws thermal energy away from targeted tissue, reducing coagulation volume [28,38]. It is highly plausible that shrinkage of perivascular tumors may be compromised in this way. Subphrenic tumors are often obscured on sonography due to overlapping lung tissue or ribs, which may hamper ablative efforts [24,39]. Nonetheless, only completely ablated tumors were allowed in this study, eliminating this possibility. In theory, continuous sliding of the diaphragm over a fixed subphrenic tumor may dissipate heat during ablative treatment, and we suspected that ventilatory convective heat loss would also come into play. Still, substantial tumor shrinkage (>30%) appears inevitable as an immediate effect of RF ablation, regardless of tumor location; and although Dodd et al. [40] have reported a significant inverse relation between the extent of RF ablation and the rate of portal venous flow, the presence of portal hypertension had no impact on degree of post-ablative tumor shrinkage in our study. Knowledge of this variability of tumor shrinkage according to tumor location would be helpful to perform RF ablation and interpret post-ablation imaging in clinical practice.

This study has a number of limitations. Its retrospective design inherently implies selection bias. Furthermore, volumetric quantification software (still a clinical scarcity) was not engaged for the measuring of tumors. However, CT slice thickness and slice interval were minimized, and the PACS magnification function aided in measurement precision. Third, in nine patients MRI scans were used instead of pre-treatment CT scans for image analysis. The difference in the modality may have influenced the perceived tumor measurement. Finally, the combined use of TACE/RF ablation was needed to clarify tumor margins on post-ablative CT studies. The diminished circulatory heat loss achieved via TACE likely served to enhance RF ablative zones [13].

In conclusion, significant tumor shrinkage occurs immediately after RF ablation in patients with HCC. Tumor location is an important factor, significantly impacting the degree of post-ablative tumor shrinkage.

## Supporting information

S1 Dataset (XLSX)

## References

[1] Forner A, Llovet JM, Bruix J (2012) Hepatocellular carcinoma. Lancet 379: 1245–1255. 10.1016/S0140-6736(11)61347-0 22353262 PMC13537732

[2] Lim HK (2000) Radiofrequency thermal ablation of hepatocellular carcinomas. Korean J Radiol 1: 175–184. 10.3348/kjr.2000.1.4.175 11752952 PMC2718198

[3] Wall MS, Deng XH, Torzilli PA, Doty SB, O'Brien SJ, Warren RF (1999) Thermal modification of collagen. J Shoulder Elbow Surg 8: 339–344. 10472007 10.1016/s1058-2746(99)90157-x

[4] Yang D, Converse MC, Mahvi DM, Webster JG (2007) Measurement and analysis of tissue temperature during microwave liver ablation. IEEE Trans Biomed Eng 54: 150–155. 10.1109/TBME.2006.884647 17260866

[5] Ganguli S, Brennan DD, Faintuch S, Rayan ME, Goldberg SN (2008) Immediate renal tumor involution after radiofrequency thermal ablation. J Vasc Interv Radiol 19: 412–418. 10.1016/j.jvir.2007.10.024 18295702

[6] Brace CL, Diaz TA, Hinshaw JL, Lee FT Jr. (2010) Tissue contraction caused by radiofrequency and microwave ablation: a laboratory study in liver and lung. J Vasc Interv Radiol 21: 1280–1286. 10.1016/j.jvir.2010.02.038 20537559 PMC2920145

[7] Sommer CM, Sommer SA, Mokry T, Gockner T, Gnutzmann D, Bellemann N, et al (2013) Quantification of tissue shrinkage and dehydration caused by microwave ablation: experimental study in kidneys for the estimation of effective coagulation volume. J Vasc Interv Radiol 24: 1241–1248. 10.1016/j.jvir.2013.04.008 23792128

[8] Rossmann C, Garrett-Mayer E, Rattay F, Haemmerich D (2014) Dynamics of tissue shrinkage during ablative temperature exposures. Physiol Meas 35: 55–67. 10.1088/0967-3334/35/1/55 24345880 PMC3924587

[9] Goldberg SN, Kamel IR, Kruskal JB, Reynolds K, Monsky WL, Stuart KE, et al (2002) Radiofrequency ablation of hepatic tumors: increased tumor destruction with adjuvant liposomal doxorubicin therapy. AJR Am J Roentgenol 179: 93–101. 10.2214/ajr.179.1.1790093 12076912

[10] Terraz S, Constantin C, Majno PE, Spahr L, Mentha G, Becker CD (2007) Image-guided multipolar radiofrequency ablation of liver tumours: initial clinical results. Eur Radiol 17: 2253–2261. 10.1007/s00330-007-0626-x 17375306

[11] Cassinotto C, Denys A, Gay F, Duran R, Hocquelet A, Piron L, et al (2018) Radiofrequency Ablation of Liver Tumors: No Difference in the Ablation Zone Volume Between Cirrhotic and Healthy Liver. Cardiovasc Intervent Radiol 41: 905–911. 10.1007/s00270-018-1898-z 29484466

[12] Kang SG, Yoon CJ, Jeong SH, Kim JW, Lee SH, Lee KH, et al (2009) Single-session combined therapy with chemoembolization and radiofrequency ablation in hepatocellular carcinoma less than or equal to 5 cm: a preliminary study. J Vasc Interv Radiol 20: 1570–1577. 10.1016/j.jvir.2009.09.003 19879777

[13] Kim JW, Kim JH, Won HJ, Shin YM, Yoon HK, Sung KB, et al (2012) Hepatocellular carcinomas 2–3 cm in diameter: transarterial chemoembolization plus radiofrequency ablation vs. radiofrequency ablation alone. Eur J Radiol 81: e189–193. 10.1016/j.ejrad.2011.01.122 21353417

[14] Yin X, Zhang L, Wang YH, Zhang BH, Gan YH, Ge NL, et al (2014) Transcatheter arterial chemoembolization combined with radiofrequency ablation delays tumor progression and prolongs overall survival in patients with intermediate (BCLC B) hepatocellular carcinoma. BMC Cancer 14: 849 10.1186/1471-2407-14-849 25409554 PMC4256894

[15] Xie H, Wang H, An W, Ma W, Qi R, Yang B, et al (2014) The efficacy of radiofrequency ablation combined with transcatheter arterial chemoembolization for primary hepatocellular carcinoma in a cohort of 487 patients. PLoS One 9: e89081 10.1371/journal.pone.0089081 24586515 PMC3930665

[16] Min JH, Lee MW, Cha DI, Jeon YH, Shin SW, Cho SK, et al (2013) Radiofrequency ablation combined with chemoembolization for intermediate-sized (3–5 cm) hepatocellular carcinomas under dual guidance of biplane fluoroscopy and ultrasonography. Korean J Radiol 14: 248–258. 10.3348/kjr.2013.14.2.248 23483753 PMC3590337

[17] Ahmed M, Solbiati L, Brace CL, Breen DJ, Callstrom MR, Charboneau JW, et al (2014) Image-guided tumor ablation: standardization of terminology and reporting criteria—a 10-year update. Radiology 273: 241–260. 10.1148/radiol.14132958 24927329 PMC4263618

[18] Nakazawa T, Kokubu S, Shibuya A, Ono K, Watanabe M, Hidaka H, et al (2007) Radiofrequency ablation of hepatocellular carcinoma: correlation between local tumor progression after ablation and ablative margin. AJR Am J Roentgenol 188: 480–488. 10.2214/AJR.05.2079 17242258

[19] Bruix J, Sherman M (2011) Management of hepatocellular carcinoma: an update. Hepatology 53: 1020–1022. 10.1002/hep.24199 21374666 PMC3084991

[20] Choe WH, Kim YJ, Park HS, Park SW, Kim JH, Kwon SY (2014) Short-term interval combined chemoembolization and radiofrequency ablation for hepatocellular carcinoma. World J Gastroenterol 20: 12588–12594. 10.3748/wjg.v20.i35.12588 25253962 PMC4168095

[21] Lee DH, Lee JM, Lee JY, Kim SH, Yoon JH, Kim YJ, et al (2014) Radiofrequency ablation of hepatocellular carcinoma as first-line treatment: long-term results and prognostic factors in 162 patients with cirrhosis. Radiology 270: 900–909. 10.1148/radiol.13130940 24475823

[22] Kim YJ, Raman SS, Yu NC, Busuttil RW, Tong M, Lu DS (2008) Radiofrequency ablation of hepatocellular carcinoma: can subcapsular tumors be safely ablated? AJR Am J Roentgenol 190: 1029–1034. 10.2214/AJR.07.2293 18356451

[23] Kang TW, Lim HK, Lee MW, Kim YS, Rhim H, Lee WJ, et al (2016) Long-term Therapeutic Outcomes of Radiofrequency Ablation for Subcapsular versus Nonsubcapsular Hepatocellular Carcinoma: A Propensity Score Matched Study. Radiology 280: 300–312. 10.1148/radiol.2016151243 26824711

[24] Kang TW, Rhim H, Kim EY, Kim YS, Choi D, Lee WJ, et al (2009) Percutaneous radiofrequency ablation for the hepatocellular carcinoma abutting the diaphragm: assessment of safety and therapeutic efficacy. Korean J Radiol 10: 34–42. 10.3348/kjr.2009.10.1.34 19182501 PMC2647171

[25] Head HW, Dodd GD 3rd, Dalrymple NC, Prasad SR, El-Merhi FM, Freckleton MW, et al (2007) Percutaneous radiofrequency ablation of hepatic tumors against the diaphragm: frequency of diaphragmatic injury. Radiology 243: 877–884. 10.1148/radiol.2433060157 17517940

[26] Kang TW, Lim HK, Lee MW, Kim YS, Choi D, Rhim H (2014) Perivascular versus nonperivascular small HCC treated with percutaneous RF ablation: retrospective comparison of long-term therapeutic outcomes. Radiology 270: 888–899. 10.1148/radiol.13130753 24475820

[27] Wong SN, Lin CJ, Lin CC, Chen WT, Cua IH, Lin SM (2008) Combined percutaneous radiofrequency ablation and ethanol injection for hepatocellular carcinoma in high-risk locations. AJR Am J Roentgenol 190: W187–195. 10.2214/AJR.07.2537 18287411

[28] Lu DS, Raman SS, Limanond P, Aziz D, Economou J, Busuttil R, et al (2003) Influence of large peritumoral vessels on outcome of radiofrequency ablation of liver tumors. J Vasc Interv Radiol 14: 1267–1274. 14551273 10.1097/01.rvi.0000092666.72261.6b

[29] Khalilzadeh O, Baerlocher MO, Shyn PB, Connolly BL, Devane AM, Morris CS, et al (2017) Proposal of a New Adverse Event Classification by the Society of Interventional Radiology Standards of Practice Committee. J Vasc Interv Radiol 28: 1432–1437 e1433. 10.1016/j.jvir.2017.06.019 28757285

[30] Lee JK, Siripongsakun S, Bahrami S, Raman SS, Sayre J, Lu DS (2016) Microwave ablation of liver tumors: degree of tissue contraction as compared to RF ablation. Abdom Radiol (NY) 41: 659–666. 27039193 10.1007/s00261-016-0725-8

[31] Horiike N, Iuchi H, Ninomiya T, Kawai K, Kumagi T, Michitaka K, et al (2002) Influencing factors for recurrence of hepatocellular carcinoma treated with radiofrequency ablation. Oncol Rep 9: 1059–1062. 12168073

[32] Wang X, Sofocleous CT, Erinjeri JP, Petre EN, Gonen M, Do KG, et al (2013) Margin size is an independent predictor of local tumor progression after ablation of colon cancer liver metastases. Cardiovasc Intervent Radiol 36: 166–175. 10.1007/s00270-012-0377-1 22535243 PMC4122121

[33] Shin S, Lee JM, Kim KW, Joo I, Han JK, Choi BI, et al (2014) Postablation assessment using follow-up registration of CT images before and after radiofrequency ablation (RFA): prospective evaluation of midterm therapeutic results of RFA for hepatocellular carcinoma. AJR Am J Roentgenol 203: 70–77. 10.2214/AJR.13.11709 24951197

[34] Iyer RS, Timm BA, Mitsumori LM, Kolokythas O (2010) Image fusion as a new postprocessing method to evaluate the radiofrequency ablation zone after treatment of malignant liver tumors. J Comput Assist Tomogr 34: 226–228. 10.1097/RCT.0b013e3181c4f797 20351510

[35] Kim KW, Lee JM, Klotz E, Kim SJ, Kim SH, Kim JY, et al (2011) Safety margin assessment after radiofrequency ablation of the liver using registration of preprocedure and postprocedure CT images. AJR Am J Roentgenol 196: W565–572. 10.2214/AJR.10.5122 21512046

[36] Passera K, Selvaggi S, Scaramuzza D, Garbagnati F, Vergnaghi D, Mainardi L (2013) Radiofrequency ablation of liver tumors: quantitative assessment of tumor coverage through CT image processing. BMC Med Imaging 13: 3 10.1186/1471-2342-13-3 23324557 PMC3626768

[37] Park J, Lee JM, Lee DH, Joo I, Yoon JH, Park JY, et al (2017) Value of Nonrigid Registration of Pre-Procedure MR with Post-Procedure CT After Radiofrequency Ablation for Hepatocellular Carcinoma. Cardiovasc Intervent Radiol. 10.1007/s00270-017-1571-y 28091728

[38] Lu DS, Raman SS, Vodopich DJ, Wang M, Sayre J, Lassman C (2002) Effect of vessel size on creation of hepatic radiofrequency lesions in pigs: assessment of the "heat sink" effect. AJR Am J Roentgenol 178: 47–51. 10.2214/ajr.178.1.1780047 11756085

[39] Song I, Rhim H, Lim HK, Kim YS, Choi D (2009) Percutaneous radiofrequency ablation of hepatocellular carcinoma abutting the diaphragm and gastrointestinal tracts with the use of artificial ascites: safety and technical efficacy in 143 patients. Eur Radiol 19: 2630–2640. 10.1007/s00330-009-1463-x 19557416

[40] Dodd GD 3rd, Dodd NA, Lanctot AC, Glueck DA (2013) Effect of variation of portal venous blood flow on radiofrequency and microwave ablations in a blood-perfused bovine liver model. Radiology 267: 129–136. 10.1148/radiol.12120486 23297326

